# Motive Power of the Blood; Experiments on an Alligator at New Orleans

**Published:** 1852-03

**Authors:** 


ARTICLE VI.

*Motive Power of the Blood—the Experiments on an Alligator at New Orleans.*

[In consequence of the suggestion contained in Mrs. Williard's letter to Dr. Cartwright (published in this Journal of January 7th,) that as some persons regarded the great alligator experiment as a hoax, it might be well for him to fortify his own testimony by that of other persons present, especially those mentioned in his letter as aiding in its performance, that eminent gentleman wrote to Drs. Dowler and Nutt, and Prof. Forshey; and having received their replies, he forwarded them to Mrs. Williard, requesting her to send them to the office of the Journal. Some necessary delay has occurred; but they arrive opportunely to satisfy Dr. Chandler and others, that however the rationale is explained, the remarkable experiment related in this Journal as truth, is so in reality.----EDITOR *Boston Med. & Surg. Jour.*]

*New Orleans, Dec.* 29, 1851.

*To Bennett Dowler, M. D.*

DEAR SIR:—1. Did or did you not perform an experiment upon an alligator, in presence of Prof. Forshey, myself and others, by tying the trachea, and returning it to its den?

2. If so, was or was it not found, some half hour afterwards apparently dead; and did or did you not have it brought from its den, into an upper story of a house on Tchoupitoulas st., laid on a table, and its viscera exposed to view by a dissection.

3. If so, did or did not the animal move or show any signs of pain during the dissection?

4. Were or were not the lungs, after this dissection, inflated by Prof. Forshey; and if so, did or did not the animal come to life?

5. If it came to life, did or did it not become so vigorous in its movements as to make it necessary to hold it or tie it; and if so, did or did you not afterwards adopt the expedient of binding it with cords to a plank to enable you to prosecute the subsequent vivisection without interruption from its movements?

By answering the above inquiries, you will oblige

Your ob't serv't,

SAMUEL A. CARTWRIGHT.

---

*New Orleans*, *Dec.* 31, 1851.

*To Samuel A. Ca twright, M D.*

Dear Sir:----I hasten to acknowledge the reception of your note (of the 29th inst.,) which I did not get until last night. On the reverse of the same sheet, I beg leave to reply to your questions, *seriatim*.

1. On the 20th of August, at 9 A. M., I tied the trachea of a healthy alligator, and returned it to its den, in your presence, as well as in the presence of five other physicians, Prof. Forshey, and others not of the profession.

2. In about thirty or forty minutes after the operation mentioned, the animal was brought from below to the third story of a house on Tchoupitoulas st., the same gentlemen being present; and the alligator appearing quite dead, was laid on the table for anatomical examination.

3. During several minutes, while I was demonstrating the viscera by dissection, the animal remained completely passive and motionless, and apparently completely dead.

4. After I had exposed to view the thoracic and abdominal viscera, I removed the ligature from, and made an opening in the trachea. Prof. Forshey having repeatedly inflated the lungs by means of a tube introduced into the opening, life gradually returned to the animal.

5. The animal's motions became vigorous, and its limbs were so well directed to the seat of the dissection, that it became necessary to hold, and finally to tie the same to a plank, in order to complete the demonstration (of the organs,) which was carried on for near two hours, with some interruptions from a simultaneous vivisection of another alligator in the same room.

The above facts, noted and recorded a few hours after their occurrence (in Vol. XIX., p. 764 MS.) were observed by numerous witnesses, as well as yourself.

I am, dear Sir, yours truly,

BENNET DOWLER.

*New Orleans, Jan.* 7, 1852.

*To Dr. C. R. Nutt.*

Dear Sir:---Please be so kind as to answer the following questions, and much oblige Your o'bt sevr't,

SAM'L. A. CARTWRIGHT.

1. Did you not witness some experiments performed by Dr. Dowler, on Tchoupitoulas street, upon a couple of alligators, wherein one of them was resuscitated by inflation of the lungs?

2. If so, did you take notes, at the time, of the phenomena observed, and will you look over those notes and say whether the alligator resuscitated appeared to be perfectly dead before the inflating process commenced? Whether *fire* was applied to it; and whether its thorax and abdomen were laid open by the scalpel so that the viscera could be seen before inflation of the lungs commenced?

3. Did life return in a doubtful way, with only feeble manifestations; or was it vigorous life, characterized by violent motions, ruled by the will?

4. Were any means used, after resuscitation, to restrain the motions of the animal? Was it held for a time, and subsequently secured by tying?

5. Were there any motions of the heart when inflation was commenced?

*Answers to the foregoing Iuterrogatories.*

1. I was present---witnessed the vivisections of Dr. Dowler upon two alligators, one of which was decapitated, and the other strangled by exposing the trachea and tying it up with a firm and strong ligature. It was afterwards resuscitated.

2. I took notes at the time, which I am unable now to find. The strangled alligator, after the application of the ligature, exhibited the ordinary appearances of suspended animation, that of entire relaxation and total loss of motion. Rigor mortis was absent at the time Prof. Forshey inserted a proper tube into the opening made in the trachea. Fire was applied to the body, without any corresponding expression of pain. Dr. Dowler had exposed the thoracic viscera before the experiment of inflation. By inflation its lungs (large air sacs,) beautifully covered with arborescent anastomoses of bloodvessels, were exhibited.

3. Upon the continued efforts of inflation for the space of two or three minutes by Prof. Forshey, the strangled alligator manifested all the signs of renewed animation and intelligence as well as sensation, so far as its motions were unimpaired by the knife.

4. It made repeated efforts to escape; and to continue the vivisections, it was found necessary to tie it.

5. I cannot speak positively of the movements of the heart, whether it was quiescent or not. C. R. Nutt.

*January* 8, 1852.

---

*Oleander, Carrollton, La., Jan.* 1, 1852.

*To Dr. S. A. Cartwright.*

Dear Sir:---Your note relating to our experiments upon two alligators, has come to hand; and I take pleasure in adding my testimony, if it be needed, to yours and others, as to the wonderful facts developed by that examination.

I made no detailed record of the experiments. The minutes were kept by our friend, Dr. C. R. Nutt, and the results were published by Bennet Dowler, in his Contributions to Physiology.*

We manacled the alligator and laid him on his back, upon the block; cut through the skin of the throat with a sharp knife, and tied a cord tightly round his wind-pipe, and then sewed up the incision, and turned him loose in his den. He exhibited but slight evidences of pain, in the process of cutting and tying up the trachea. Very little blood was expended.

We then passed into another story of the building, and commenced the experiments on alligator No. 2.

At the lapse of twenty minutes by the watch we returned to see our first object; and, to our astonishment, found him *stone dead*. We took him from the cage, laid him upon the table and handled him, finding him lifeless and limber. We touched fire to him (as in other cases of decapitation) to which he showed no response, or motion of any kind. It was a subject of some merriment, that "to kill an alligator, cutting his head off, his heart and lungs out, and probing his spinal mar-

* We omit here a part of Prof. Forshey's letter, as it refers not to facts, but to the peculiar views of Dowler who was experimenting on alligators for a different object from that which induced Prof Forshey and Dr. Cartwright to attempt the resuscitation described. A forthcoming work of Dr. Dowler, said to be of great interest, and much originality, will give some new phases of this experiment, and detail another made upon a second alligator. Some months elapsed before Dr. Cartwright wrote his description to Mrs. Williard, as he waited to give Dr. Dowler the advantage of being first to produce these singular experiments.

row, were of little use; but that a few minutes choking was effectual." We cut loose the ligature of the trachea, and Dr. Dowler commenced dissecting about the throax; but still no signs of life appeared.

It was at this stage of our inquisition, that I requested you and Dr. Dowler to await an experiment to resuscitate him, by inflating his lungs. It was thought scarcely worth the time; and as some delay occurred in my search for a proper tube,* the knife had severed his ribs from the sternum when I commenced inflating the lungs. I injected the air with all my power, and then expelled it by compression, and repeated this several times, when signs of life appeared. And in two or three minutes more, about six or seven minutes in all, he was wide awake and ready to defend himself.

After this I do not remember the order of successive experiments; but I know that, to the astonishment and satisfaction of every one present, his re-animation was complete, and his subsequent actions as intelligent, and nearly as powerful, as before he was throttled, and that his subsequent death was produced by being again manacled, and having his heart and lungs dissected out.

Curious and profoundly interesting as this series of experiments were to all of us, and must be to every reflecting mind—especially in their psychological bearings---I regard this one of resuscitation after death, by inflating the lungs, as having a directly practical use, of far more ready and general application than any one of the series.

The subject was not then new to me, as a mere random thought thrusting itself up accidently. In the year 1838, I witnessed the sudden death of a most valued friend. The cause of death was such that my mind never became reconciled to it. It was too late when the suggestion came; but it forced itself upon me to conviction----that had the lungs been inflated, after the cause of death was removed, re-animation would have followed. My professional pursuits were not such as to afford me an opportunity to make such experiments upon human life; but I frequently spoke of it to physicians, yet never met an opportunity to test it upon a life of any kind, until on this occasion.

* Dr. Cartwright in a letter remarks that these removals and delays, together with vivisection, consumed so much time, that fully an hour occurred from the throttling to the resuscitation.

Let me then state the practical lesson which results from this experiment, and indulge the hope that your professional brethren will fully test its value.

"When death results from a cause, which can readily be removed, after death re-animation may be effected, and the machinery of life set in motion, by artificially inflating of the lungs." I have the satisfaction to remain

Your faithful friend,

CALEB G. FORSHEY.

—*Boston Med. and Surg. Jour.*

